# The Role of Skunks in the Epidemiology of Rabies in the State of Yucatan from 2000 to 2022: Current Perspectives and Future Research Directions

**DOI:** 10.3390/microorganisms13020262

**Published:** 2025-01-25

**Authors:** Paola Puebla-Rodríguez, Octavio Patricio García-González, Rocío Sánchez-Sánchez, Mauricio Díaz-Sánchez, Juan Carlos Del Mazo, Isaías Sauri-González, Adriana Alonzo-Góngora, Gabriel García-Rodríguez, Irma López-Martínez, Nidia Aréchiga-Ceballos

**Affiliations:** 1 Instituto de Diagnóstico y Referencia Epidemiológicos, Dirección General de Epidemiología, Secretaría de Salud, Ciudad de México 01480, Mexico; pueblapao@gmail.com (P.P.-R.); juan.delmazo@salud.gob.mx (J.C.D.M.); irma.lopez@salud.gob.mx (I.L.-M.); 2 Facultad de Medicina Veterinaria y Zootecnia, Universidad Nacional Autónoma de Mexico, Ciudad de México 14510, Mexico; 3 Instituto Traslacional de Singularidad Genómica, Irapuato 36615, Mexico; octavio@itrasig.org (O.P.G.-G.); tiemporeal@itrasig.org (R.S.-S.); direcciongeneral@itrasig.org (M.D.-S.); 4 Laboratorio Central Regional de Mérida, Comité Estatal para el Fomento y Protección Pecuaria del Estado de Yucatán S.C.P., Mérida 97130, Mexico; sauri_san@yahoo.com (I.S.-G.); abag_1970@yahoo.com (A.A.-G.); 5 Dirección General de Epidemiología, Secretaría de Salud, Ciudad de México 01480, Mexico; rogamed@icloud.com

**Keywords:** *Spilogale yucatanensis*, *Cytochrome B*, rabies virus, reservoirs

## Abstract

In 2019, the World Health Organization (WHO) and the Pan American Health Organization (PAHO) bestowed upon Mexico the status of a country free from canine-transmitted human rabies; however, the lingering public health challenge in the nation continues to be the sylvatic cycle of rabies. In Mexico, skunks play a significant role as reservoirs for four antigenic variants of the rabies virus. Nonetheless, up to this point, the specific skunk species involved in this cycle had not been accurately established. This study (2002–2022) aims to identify the taxonomy of skunk specimens diagnosed as rabies-positive in the state of Yucatan, in order to determine the species that serves as the reservoir for the Yucatan sylvatic lineage of rabies. This was achieved through the sequencing of an approximately 680 bp fragment of the mitochondrial gene *cytochrome B*. All samples belong to the species *Spilogale angustifrons yucatanensis*. This discovery offers valuable information for understanding and managing the wildlife rabies cycle in Mexico. It also contributes to an improved understanding of the taxonomy within the genus *Spilogale.*

## 1. Introduction

Rabies is caused by neurotropic viruses of the genus *Lyssavirus* in the family *Rhabdoviridae*. It is transmissible to all mammals and it is almost uniformly fatal [1]. Contact with infected saliva through a bite from a rabid animal is the main route of rabies virus (RABV) infection in humans [2], although it can also occur through contact with infected saliva in open wounds or mucous membranes [3].

Rabies remains a zoonosis of great public health importance, present in 150 countries and causing around 59,000 annual deaths, primarily in Asia and Africa, usually following bites from infected dogs [4]. Mexico successfully controlled human rabies transmitted by dogs in 2006, but the disease remains a challenge due to the presence of other wild mammals that can transmit it to humans and domestic animals [5,6,7]. A large part of Mexican biodiversity has a restricted distribution. Additionally, there is a high number of endemic species due to the diverse geography, climates and types of vegetation, which facilitate the species’ adaptation to specific environmental conditions [8,9].

It has been documented that several carnivore species could act as RABV transmitters and reservoirs, including striped skunks (*Mephitis mephitis*), spotted skunks (*Spilogale putorius*), hooded skunks (*Mephitis macroura*), hog-nosed skunks (*Conepatus leuconotus*) and gray foxes (*Urocyon cinereoargenteus*) [7,10,11,12,13].

To facilitate the understanding of the epidemiological scenario of rabies, two key concepts have been defined: “reservoir” and “vector”. A reservoir is a species that can maintain the rabies virus circulating in nature without the need for other host species and that preserves its own antigenic variant or genetic lineage. On the other hand, a vector is a host species that can transmit the virus but does not maintain it circulating independently in nature, and that is not associated with any specific variant or lineage [14].

Skunks, from the family Mephitidae, consist of four genera and 11 species that are globally recognized, characterized by anal odoriferous glands and black-and-white fur patterns [15,16,17]. The geographical distribution of skunk species is not well known, and there are few molecular identification studies [18]. They are almost exclusively found in the Americas, with three genera and nine species, ranging from Canada to Chile [16,19,20].

**Figure 1:**
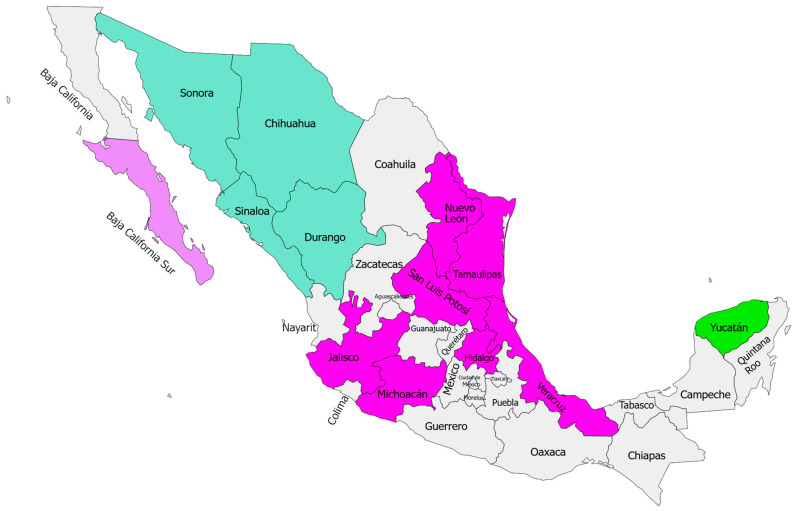
Map of the Mexican Republic showing the distribution of the four rabies virus variants related to skunks. RABVV 10 light purple, RABVV 1 in green, RABVV 8 in magenta, and atypical RABVV Yucatan sylvatic in lime green.

Skunks are recognized as major rabies transmitters in the United States of America (USA) and Mexico, but specimens sent to laboratories for diagnosis are often not correctly identified [7,21]. In Mexico, at least four rabies virus variants (RABVV) related to skunks have been identified (Figure 1). RABVV 10, restricted to Baja California Sur, has the skunk *Spilogale putorius lucasana* as its reservoir and is related to the California variant circulating in skunks, although recent studies show they belong to distinct evolutionary lineages [22,23]. In the north, the RABVV1 “atypical” skunk variant, originating from canids but maintained in skunk populations without species identification, has been reported.

In the states of Hidalgo, Jalisco, Nuevo Leon, San Luis Potosi, Tamaulipas, and Veracruz, RABVV8 has been isolated in spotted skunks (*S. putorius*) and striped skunks (*Conepatus leuconotus*) [12,23,24]. Unlike other variants, RABVV8 has a chiropteran origin, possibly from hematophagous bats [12,23,24]. However, the Yucatan sylvatic variant, described in 2017, so far, appears to be restricted to the state of Yucatan. This variant has been isolated in both domestic and wild animals such as dogs (*Canis lupus familiaris*), cats (*Felis catus*), white-nosed coatis (*Nasua narica*), and tepezcuintles (*Cuniculus paca*) [6,25,26]. The evidence suggests that skunks are the main reservoirs of this variant, although it has not been determined which of the two skunk species in Yucatan (*Spilogale angustifrons* or *Conepatus semistriatus)* is responsible for actively maintaining and transmitting this RABVV [25,27].

Due to their high susceptibility and long incubation periods, skunks are known for their role in rabies transmission to humans; however, specific knowledge about the reservoir species in Mexico is still lacking. In addition, some skunk species can live near urban areas, increasing the risk of transmission to human populations [7,11,28].

Mitochondrial DNA sequence analyses have shown that skunks form a monophyletic group within the Mephitidae family, which separated from musteloids before the emergence of the Procyonidae and Mustelidae families [29]. Among the 11 currently recognized skunk species, molecular studies have mainly focused on understanding relationships within genera, especially *Conepatus* [16,17,18]. Phylogenetic studies using cyt *b* nucleotide sequences revealed that *Mephitis* and *Spilogale* share a common ancestor, separating after *Conepatus*. These studies also provided insights into species relationships within *Spilogale*, such as *S. gracilis* vs. *S. putorius* [30]. Despite these findings, the phylogenetic relationships among these families remain uncertain, highlighting the need for further systematic studies [17].

The taxonomy of *Spilogale* has evolved and has been controversial due to the difficulty in distinguishing species based on external morphology. Initially, only two species were recognized, but taxonomic revisions increased this number to 14, with the currently accepted number being four [31,32,33]. These changes were based on cranio-dental features, pelage patterns, and geographic distribution, but the external morphology is highly conserved, complicating classical taxonomy [34,35].

Using DNA sequencing and phylogenetic analysis, molecular studies within *Spilogale* have focused on analyzing genetic diversity and evaluating phylogenetic subdivisions and subspecies boundaries [18,31,32,33].

One of the four *Spilogale* species, *S. angustifrons*, described in 1902, was excluded from valid species lists for about 90 years due to reliance on morphological characteristics. Its reinclusion took place in 1996 through molecular techniques, confirming its distinct status as *S. angustifrons*, and it is now recognized from central Mexico to Costa Rica [16,36,37,38].

A recent study by McDonough et al. (2022) presented evidence that the taxonomic diversity of *Spilogale* is more extensive than previously documented; it identified seven species instead of four [16,17]. The study confirmed the distribution of *S. angustifrons* in Mexico and proposed a new endemic species in Yucatan, *Spilogale yucatanensis*, previously considered a variant of *S. angustifrons.*

As mentioned above, the objective of this study was to standardize the PCR reactions for the molecular taxonomic identification of skunk specimens diagnosed as RABV-positive in Yucatan, to determine the skunk species that serves as the reservoir for the “Yucatan sylvatic” lineage. The standardization was performed on nervous tissue, which is a contribution to rabies virus surveillance, as no additional tissue is needed beyond that which is routinely received for rabies diagnosis.

## 2. Materials and Methods

### 2.1. Samples Selection

All brain tissues from rabies cases in skunks from Yucatan were obtained from the sample bank of the Rabies Laboratory at InDRE and the Merida Regional Central Laboratory from the State Committee for the Promotion and Protection of Livestock of the State of Yucatan from 2000 to 2023. As controls for the cyt *b* tests, skunks from the states of Chihuahua and Baja California Sur (BCS), were used. These regions were selected due to the absence of Yucatan skunk distribution, according to CONABIO acronym of Comisión Nacional para el Conocimiento y Uso de la Biodiversidad, (in English: National Commission for the Knowledge and Use of Biodiversity) data (Table 1).

### 2.2. Diagnosis and Antigenic Characterization of the Rabies Virus

All samples from Yucatan, Chihuahua, and Baja California Sur underwent diagnosis using the fluorescent antigen test (FAT), with subsequent antigenic characterization employing a reduced panel of eight monoclonal antibodies (MAbs) standardized by the Center for Disease Control and Prevention (CDC). This panel is capable of identifying 11 reactivity patterns associated with various mammals involved in rabies virus maintenance and transmission in the Americas [39].

Antigenic characterization was directly applied to brain smears, using impressions on an eight-well slide with a 6 mm diameter. Following the technique outlined by Jaramillo Reyna and colleagues in 2020, positive reaction for each monoclonal antibody (20 μL) was determined if more than 50% of the fluorescing foci exhibited a brilliant apple green color. The purpose was to ascertain that the antigenic profile did not correspond to any documented patterns within the designated panel, thereby accurately classifying them as “atypical” samples.

### 2.3. Genetic Characterization of the Rabies Virus

Following the manufacturer’s instructions, the genetic material was extracted from all brain tissue samples utilizing the QIAGEN^®^ “QIAamp Viral RNA” (QIAGEN, Hilden, Germany) commercial kit. RT-PCR was employed to amplify a specific nucleoprotein region using the following primers: 550 FW (5′ATG TGY GCT AAY TGG AGY AC 3′) [40] and 304 RABV (5′ TTG ACG AAG ATC TTG CTC AT 3′) [41]. Amplification products and partial sequencing procedures were conducted by the methods detailed by Garcés-Ayala et al. (2017) [6].

### 2.4. Genetic Characterization of Skunk Species

Instructions included in the QIAamp DNA mini kit from QIAGEN^®^ (Hilden, Germany) were followed in order to obtain mitochondrial DNA from all the available samples (brain tissues).

#### 2.4.1. Design of Specific Oligonucleotides

Three pairs of specific oligonucleotides were designed for the cyt *b* gene of three skunk genera: *Spilogale, Conepatus*, and *Mephitis*. These oligonucleotide sets were based on sequences obtained from the GenBank (https://www.ncbi.nlm.nih.gov/genbank/ accessed on 30 July 2023) of the respective species, with a preference for full genome sequences of cyt *b*. Computational analysis (in silico) was conducted to design oligonucleotides, allowing selective amplification of the target species. This process involved sequence visualization using the Ugene program V47.0 [42] with the selection of the most conserved regions of the cyt *b* gene for the skunk species of interest. An analysis utilizing tools such as BLAST and Primer-BLAST was performed to ensure that the oligonucleotide designs were suitable for species detection. The sequences of the designed oligonucleotides were synthesized at the T4 oligo laboratory (https://t4oligo.com) (accessed on 8 August 2023) and are detailed in Table 2.

#### 2.4.2. Standardization of Polymerase Chain Reaction (PCR) for Genetic Characterization of Skunks’ Species

The technique was carried out using the commercial kit “Taq DNA Polymerase” from Roche (Mannheim, Germany). The necessary volume of the master mix was prepared for the number of samples to be processed. The reaction is detailed in Table 3. In total, 25 µL of the master mix and 5 µL of the corresponding DNA template were placed in each tube. The tubes were then placed in the thermocycler to start the run, using the protocol described in Table 4.

### 2.5. Sequencing and Phylogenetic Reconstruction of the Rabies Virus

Once the run was completed, the visualization of the products was carried out following the manufacturer’s instructions using the “Bioanalyzer 2100 Agilent” (Waldbronn, Germany) equipment and the Agilent “DNA Chip 7500” (Winooski, VT, USA) reagent kit.

The sequencing of rabies virus was conducted at InDRE following the methodology described by Garcés-Ayala et al. (2022) [43].

As soon as the results were obtained, the sequences were edited and manually corrected using the BioEdit program V.5.0.9 [44]. Subsequently, a BLAST analysis was performed on the NCBI website (https://blast.ncbi.nlm.nih.gov/Blast.cgi) (accesed on 30 August 2023) to identify the similarity percentage of the sequences obtained in this study compared to the data registered in the database.

A multiple alignment of the sequences was performed using MEGA 11 11.0.13 software with the MUSCLE algorithm [45,46]. The most appropriate evolutionary model for the analysis of the rabies virus was identified as the GTR model.

A phylogenetic analysis was conducted focusing on sequences of a fragment of the nucleoprotein of the virus in order to identify variants of the rabies virus in mammals that are part of the terrestrial cycle in the state of Yucatan. This analysis used rabies virus sequence data from Mexico, North America, and Central America, as well as sequences generated in this project (Supplementary Table S1). The phylogenetic tree was constructed using the Maximum Likelihood method, with 1000 bootstrap replicates to strengthen the robustness of the results. MEGA 11 software V11.0.13was employed for tree construction, and subsequent modifications were made using Inkscape software V 1.4 [47]

### 2.6. Sequencing and Phylogenetic Reconstruction of Skunks Species

We proceeded as previously described in this section in order to visualize the PCR products, but in the case of the DNA samples obtained from the nervous tissues, these products were sent for sequencing at the Translational Genomic Singularity Institute (ITRASIG by its acronym in Spanish) Irapuato, Guanajuato, Mexico.

The DNA sequences were edited and manually corrected using the BioEdit program [44]. A multiple alignment of the sequences was performed using MEGA 11 software V. 11.0.13 with the MUSCLE algorithm [45,46]. The most appropriate evolutionary model for the skunk analyses was identified as the Tamura 3 model.

A phylogenetic analysis was conducted focusing on mitochondrial DNA sequences or *cyt b* gene sequences of skunks, using data registered in GenBank corresponding to the eight species previously described for Mexico, as well as sequences generated in this study. Subsequently, a specific analysis was carried out with the skunk sequences belonging to the genus *Spilogale*, using mitochondrial DNA sequences or cyt *b* gene sequences available in GenBank, along with sequences generated in this project. The phylogenetic trees were constructed using the Maximum Likelihood method, with 1000 bootstrap replicates to ensure the robustness of the results. MEGA 11 software V.11.0.13 was used for tree construction, and subsequent modifications were made using Inkscape software V 1.4 [47].

### 2.7. Genetic Distance Analysis of the Cyt b Sequences

To support the data that were obtained, a genetic distance analysis was conducted to evaluate the genomic relationships among *Spilogale* skunk sequences in MEGA 11 software V.11.0.13. Genetic divergence percentages were calculated using Kimura’s two-parameter nucleotide substitution model. This analysis provided insights into the evolutionary differences among the analyzed skunk populations included in this study, establishing their genetic variability and confirming the separation between the cyt *b* sequences of *S. angustifrons* and *S. a. yucatanensis*.

## 3. Results

### 3.1. Rabies Virus Diagnosis and Antigenic Characterization

The five original brains from skunks in the state of Yucatan were confirmed as positive by FAT. Upon analyzing the samples using the reduced panel of monoclonal antibodies, it was observed that sample 3213MxskkYuc02 exhibited an antigenicity pattern that matched the pattern described for RABVV1 (Table 1). In contrast, all the other samples exhibited similar antigenicity patterns among themselves but did not match any established antigenic variant patterns in the panel; therefore, they were diagnosed as atypical.

#### Rabies Virus Genetic Characterization

**Figure 2:**
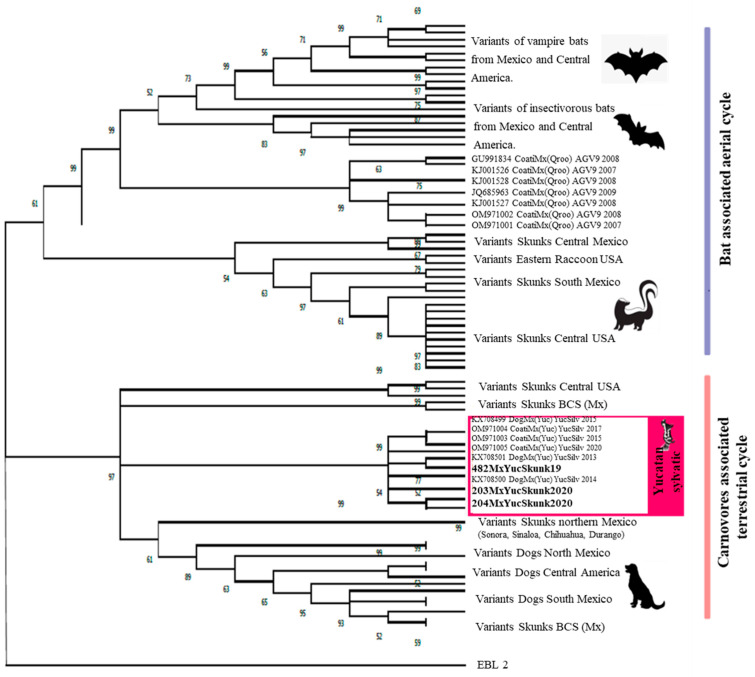
Phylogenetic analysis for the RABV. The Maximum Likelihood method was utilized with the general time-reversible model and a discrete gamma distribution, incorporating 1000 bootstrap replicates to enhance the robustness of the results. This analysis used 90 partial sequences (900 bp) of the nucleoprotein gene from several rabies virus variants circulating in the Americas. The sequences generated in this work are indicated in bold.

The phylogenetic analysis for the RABV (Figure 2) was concordant with the antigenic characterization. There is a clear separation in the grouping of the sequences. At the top of the phylogenetic tree, all the variants associated with the aerial cycle are clustered, including the strains present in skunks from central Mexico and those related to the aerial cycle in the USA. Conversely, within the clade designated for rabies variants associated with the terrestrial mammal cycle, the group of sequences corresponding to the canine RABVV is observable. A notable example is the sequences derived from dogs, classified as RABVV1. Within this clade, a well-defined subgroup includes the sequences of skunks studied in this project and those previously reported in GenBank carrying the Yucatan Sylvatic variant.

### 3.2. Skunk Genetic Characterization

**Figure 3:**
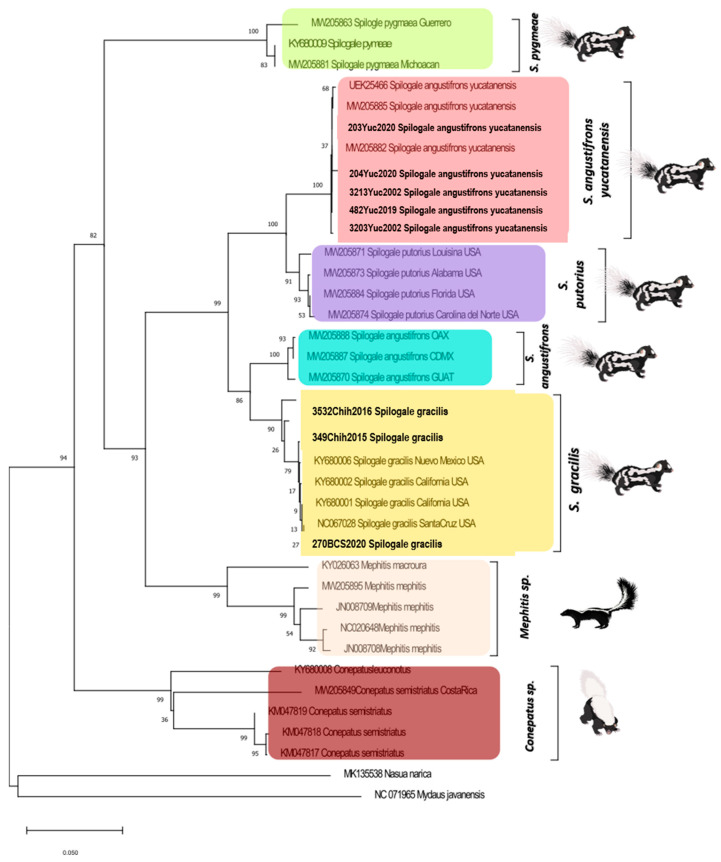
The Maximum Likelihood method was utilized with the Tamura 3 model and a discrete gamma distribution, incorporating 1000 bootstrap replicates to enhance the robustness of the results. This analysis used 45 partial sequences (680 bp) of the cyt *b* gene from the eight species of skunks present in Mexico, belonging to the genera *Mephitis, Spilogale*, and *Conepatus*. The sequences generated in this work are indicated in bold.

The phylogenetic tree was constructed using sequences from the eight species of skunks present in Mexico, belonging to the genera *Mephitidae, Spilogale*, and *Conepatus* (Figure 3). There is a clear distinction among the eight species, where the grouping of sequences belonging to each skunk species clearly defines the expected clades. In this phylogenetic analysis, a marked separation between the sequences of *S. angustifrons* and the sequences of *S.a. yucatanensis* was evident. The second tree confirmed the distinction observed in the previous analysis. In this case, only sequences from skunks of the genus *Spilogale* were used, reaffirming the separation between the sequences of *S. angustifrons* and the sequences of *S.a. yucatanensis.*

**Figure 4:**
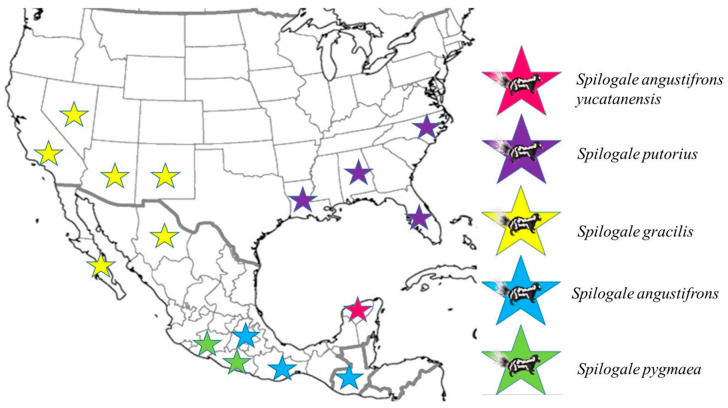
Map including sampling localities where the sequences of the *Spilogale* species analyzed in this study were reported.

To supplement the geo-epidemiological data, a map was created to visualize the geographic locations where the sequences analyzed in this project were reported (Figure 4).

#### Genetic Distance Analysis

**Figure 5:**
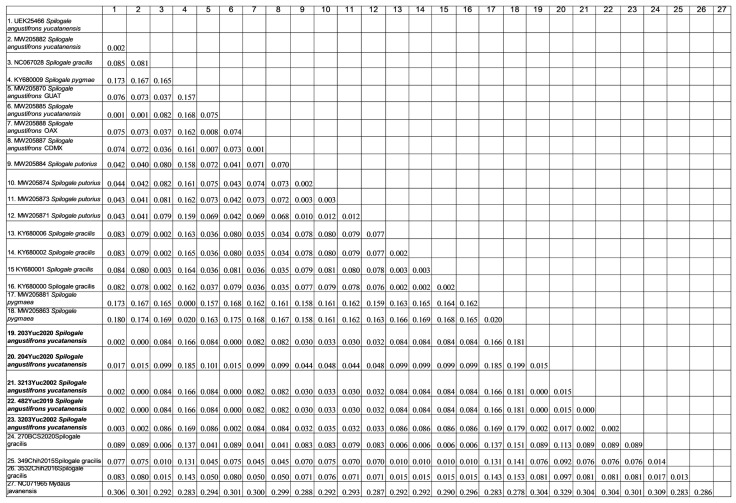
Genetic divergence percentages of the cyt *b* gene within the genus *Spilogale*, including the sequences generated in this study (indicated in bold).

The genetic divergence percentages of the samples were calculated using Kimura’s two-parameter nucleotide substitution model. The results revealed that the divergence of the cyt *b* gene within the genus *Spilogale* ranged from 0.1% to 18%. Additionally, the mean distance between the species *S. angustifrons yucatanensis* and *S. angustifrons* was determined to be 8.5%. The mean distance between the species *S. angustifrons yucatanensis* and *S. putorius* was 4.6%, while the distance between *S. angustifrons* and *S. putorius* was 6.3% (Figure 5).

## 4. Discussion

The identification of skunk species involved in rabies transmission remains challenging due to limited knowledge and complex ecological interactions, such as sympatry. Mexico has the highest level of skunk richness worldwide, including endemic species (e.g., *S. pygmae*), which is relevant to identifying suitable habitats and establishing priority conservation areas for species [20].

In North America, *Mephitis mephitis* is considered an important vector of the rabies virus [48,49,50,51,52,53]. In contrast, other skunk species do not play a significant role as reservoirs of this virus [14], and there are few or no documented cases involving other species [12,21,51,54]. In the context of Mexico, the situation is different. While in the state of Nuevo Leon, the importance of *Mephitis macroura* as a vector of the rabies virus has been demonstrated, in most of the country, it is the genus *Spilogale* that plays a crucial role as a reservoir of the virus. Several authors have mentioned that the four skunk species belonging to the genus *Spilogale*, along with two subspecies, play a role in rabies transmission in Mexico. These species include *Spilogale putorius leucoparia, Spilogale putorius lucasana, Spilogale gracilis, Spilogale pygmea*, and *Spilogale angustifrons*. There is very little evidence that *Mephitis* and *Conepatus* are significant carriers of the RABV [12,13].

In previous works that identified species based on morphology, *S. putorius* was described as the species responsible for acting as a rabies virus reservoir in Mexico [22,23]. However, the geographical distribution of this species in Mexican territory, according to CONABIO, is very limited. Some authors have even questioned the presence of this species in Mexico [16], raising certain questions about its predominant role in rabies transmission.

The genus *Spilogale* presents notable taxonomic complexity, and its evolutionary history is still not entirely clear, partly due to the limited number of specimens available for study; this situation has led to a certain confusion in species identification.

At the beginning of this project, the hypothesis was formulated that the species *S. angustifrons* could act as the natural reservoir of the Yucatan sylvatic lineage of the RABV. This hypothesis is largely based on the particular habits of this species, making it prone to playing a central role in the transmission of the disease. In comparison, the other skunk species present in the state of Yucatan, *C. semistriatus*, has certain characteristics that influence the dynamics of rabies transmission within the genus [55]. For example, the lower population density detected in these species may result in reduced exposure to the rabies virus, as there would be fewer opportunities for the virus to spread among individuals of the same species. However, more studies are needed to understand the factors that promote rabies outbreaks in skunks [56,57].

According to current reports, in the state of Yucatan, the distribution of the genus *Spilogale* is limited to a single species, the *S. angustifrons* [27]. This relationship between geographical distribution and the results of this study, in which all the skunks analyzed from the state of Yucatan belong to this species, provides, for the first time, evidence that allows us to consider *S. angustifrons* as the species responsible for maintaining and transmitting the RABV in the state of Yucatan.

*S. angustifrons* are commonly found in forested areas in the Yucatan Peninsula. They tend to inhabit regions with dense vegetation, which provides cover and hunting opportunities. While they can occasionally be found in rural areas, their abundance is generally higher in forested landscapes. The few records of *S. angustifrons* in this region may be due to the lack of mastozoological surveys in the region; therefore, the population and ecological aspects of the species remain unknown [58,59].

The RABV identified as the Yucatan sylvatic lineage, described in 2017, has significantly affected public health, having been detected in both wild and domestic animals in the state of Yucatan [6,25,26]. According to Garcés-Ayala and collaborators (2017) [6], all the domestic animals identified with this variant had a history of aggression by skunks. This suggests that skunks could play a crucial role as vectors and potential reservoirs of the Yucatan sylvatic lineage, although no taxonomic identification was performed. Further studies involving samples with atypical antigenic characterization revealed RABV sequences clustering in the Yucatan sylvatic lineage clade, indicating that this virus remains present in the region [25,26]. However, the natural reservoir of the Yucatan sylvatic lineage has not yet been definitively identified.

The sample 3213MxskkYuc02, identified as RABVV1 during antigenic characterization, with the domestic dog as its natural reservoir, is particularly significant. This variant is considered “under control” in Mexico, thanks to intensive vaccination campaigns since the 1990s, which have resulted in no recorded cases of infected dogs since 2005 or humans since 2006 [7]. Unfortunately, the poor preservation of this sample prevented its sequencing, thereby hindering the confirmation of the virus characterization and the possibility of conducting evolutionary studies to demonstrate that RABVV1 is an ancestor of the Yucatan sylvatic lineage.

To strengthen these results, phylogenetic analyses were conducted in our study. The results reveal the presence of two genetic lineages, coinciding with previous descriptions [18]. These lineages are the Western *Spilogale*, including *S. angustifrons* and *S. gracilis*, and the Eastern *Spilogale*, including *S.a. yucatanensis* and *S. putorius*. The sequences of *S. a. yucatanensis* do not isolate within the Western lineage alongside *S. angustifrons*, as would be expected, since it was previously recognized only as a subspecies.

According to the BLAST analyses performed, it was found that the five sequences of the Yucatan skunks analyzed in this project show similarities ranging from 98.8% to 100% with the previously considered subspecies *S. angustifrons yucatanensis*. This finding underscores the close genetic relationship between the skunks studied and this particular subspecies.

Additionally, it could be inferred that these lineages have evolved separately due to fragmentation events and/or ecosystem loss or biogeographic barriers [18,60]. This observed diversity could be related to the distinctive characteristics of the *Spilogale* genus, such as its small size, limited dispersal capacity, and high mortality rate, as demonstrated by various previous studies [61,62,63]. This phenomenon is similar to what is observed in other small mammals, such as bats [64].

The sequences of the skunks described in this project cluster with previously registered sequences in GenBank, which provides solid support for their identification as belonging to the subspecies *S. angustifrons yucatanensis*. It is widely accepted that a significant portion of Mexico’s biodiversity exhibits a restricted geographic distribution and can only be found in specific geographic regions, which is related to the presence of a high number of endemic species in the country [9,13]. One of the clearest examples of this assertion is the state of Yucatan [65].

In 2022, McDonough and collaborators [18] reported sufficient evidence to accept the existence of an endemic skunk species in the state of Yucatan, *S. yucatanensis* (referred to by this name from now on), although it has so far been classified as a subspecies of *S. angustifrons*. The data generated in our study strengthen the theory proposed by McDonough and her collaborators.

In our analysis, a clear distinction is demonstrated between the sequences grouped in the *S. angustifrons* clade, including sequences from locations other than Yucatan (Mexico City, Oaxaca, and Guatemala), and the sequences of all the skunks from the state of Yucatan, which include the five skunks included in this project, which are grouped together in the “*S. yucatanensis*” clade. This indicates the presence of two separate evolutionary lineages, evidencing distinct species.

To further our knowledge of the *Spilogale* genus, a genetic divergence analysis was carried out in this study. A significant difference of 8.5% was observed between *S. yucatanensis* and *S. angustifrons*, a percentage higher than that found in a previous study conducted by Dragoo and collaborators in 1993 [30]. These results support the notion that *S. yucatanensis* should be considered an independent species within the *Spilogale* genus.

Considering the distribution reported so far for the species *S. yucatanensis*, which appears to be restricted to the state of Yucatan, we identify an important factor that could explain why the Yucatan sylvatic lineage has not been identified yet in the states of Campeche and Quintana Roo, despite also being part of the Yucatan Peninsula. This reinforces the hypothesis that this skunk species could be the natural reservoir of this variant. Additionally, to enrich the data on the species, future research should focus on describing the distribution range of *S. yucatanensis* through intentional searches in the states of Campeche and Quintana Roo to determine whether it is an endemic species of the entire Yucatan Peninsula or just of the state of Yucatan.

This project also generated partial results concerning the wild-living rabies cycle panorama in other regions of Mexico. In the context of Baja California Sur, the presence of the RABVV 10 variant has been identified as being restricted to this state, where its existence in skunk populations has been confirmed. The literature has attributed the status of the natural reservoir for this variant to *S. putorius* [22,23,66,67,68].

However, current data provided by CONABIO do not corroborate the presence of this species in the state. Significantly, the results obtained from sample 70MxskkBCS20 reveal that the genetic sequence clustered with skunks of the species *S. gracilis*. This is in contrast to the information from CONABIO, which documents the presence of this species in the state of Baja California Sur. These findings constitute initial indications that could lead to the identification of the specific reservoir for the RABVV10 in question. The need for future research focusing on more robust studies to validate and consolidate this emerging hypothesis is emphasized.

Similarly, the so called “RABVV1 atypical”, previously documented in the states of Chihuahua, Sonora, Sinaloa, and Durango, has been associated with the presence of *S. putorius* according to the literature [12,22,66]. Nevertheless, the genetic sequences generated from skunks in the state of Chihuahua as part of this project show a group in the clade corresponding to the species *S. gracilis*. It is worth noting that, according to data from the epidemiological bulletin of the General Direction of Epidemiology of the Ministry of Health of Mexico, the state of Chihuahua has the highest incidence of human rabies cases transmitted by skunks. This fact underscores the relevance of undertaking more detailed studies to understand the interaction between the RABVV, the skunks, and the transmission cases in these states. Even records of domestic animals infected with a skunk variant serve to highlight the relevance of skunks in the context of public health [69].

Molecular taxonomic research using advanced genetic and molecular techniques is needed in order to refine the systematics at the species and subspecies level and determine the genetic diversity within populations, particularly for endangered species or those inhabiting fragmented habitats.

## 5. Conclusions

This study introduces a novel method for the simultaneous surveillance of the rabies virus and identification of skunk species using a single tissue (brain), advancing our understanding of the taxonomy of rabies virus reservoirs and the epidemiology of rabies in Mexico.

Our results reveal that all the rabies-positive individuals analyzed from the state of Yucatan belong to the recently proposed species, *Spilogale yucatanensis*. Additionally, the described distribution of *S. yucatanensis* provides a likely explanation for why the Yucatan sylvatic lineage is endemic to this state. This highlights the necessity of implementing rabies control measures tailored to this region. These findings offer critical information that will aid in the control and prevention of rabies.

Furthermore, our study emphasizes that researching zoonotic diseases, such as rabies, is fundamental not only for public health, but also for advancing scientific knowledge of host taxonomy and evolutionary biology.

## Figures and Tables

**Table 1:** Samples of skunks analyzed in this project.

No. InDRE	Year	Institution	Municipality	State	Origin Diagnosis	Antigenic Variant	GenBank Access Rabies Virus	GenBank Access Cyt *b*
3203MxskYuc02	2002	State Public Health Laboratory of Yucatan	ND	Yucatan	Positive	Atypical	NP	PV012461
3213MxskYuc02	2002	State Public Health Laboratory of Yucatan	ND	Yucatan	Positive	RABVV1	NP	PV012462
482MxskYuc19	2019	State Public Health Laboratory of Yucatan	ND	Yucatan	Positive	Atypical	PQ963930	PV012463
203MxskYuc20	2020	State Public Health Laboratory of Yucatan	Kinchil	Yucatan	Positive	Atypical	PQ963931	PV012464
204MxskYuc20	2020	State Public Health Laboratory of Yucatan	Kinchil	Yucatan	Positive	Atypical	PQ963932	PV012465
270MxskBCS20	2020	State Public Health Laboratory of BCS	ND	Baja California Sur	Positive	RABVV10	NP	PV012466
349MxskChih15	2015	State Public Health Laboratory of Chihuahua	ND	Chihuahua	Positive	RABVV1-“ Atypical”	NP	PV012467
3124MxskChih16	2016	State Public Health Laboratory of Chihuahua	ND	Chihuahua	Positive	RABVV1-“ Atypical”	NP	PV012468

ND: No data available. NP: Not performed.

**Table 2:** The sequences of the designed oligonucleotides.

Genus	Oligo	Sequence		Expected Molecular Weight
*Spilogale*	Sa FW	5′ TCAWCATGATGAAACTTCGGTTCC 3′	Fw	678 bp
*Spilogale*	Sa RV	5′ CCTGTTTCATGAAGGAACAGTAAATG 3′	Rv	
*Conepatus*	Cs FW	5′ GCTCTCTACTCGGAAyCTGC 3′	Fw	679 bp
*Conepatus*	Cs RV	5′ TCGTGTAGGAATAATAGGTGGAC 3′	Rv	
*Mephitis*	Mm FW	5′ TCAwCATGATGAAACTTCGGTTCC 3′	Fw	678 bp
*Mephitis*	Mm RV	5′ CCTGTTTCATGTAGGAAATAGTAAGTG3′	Rv	

**Table 3:** PCR protocol used for the cyt *b* gene of skunks.

Reagent	Volume
Buffer Mix	5 µL
Taq DNA Polymerasa	1 µL
Primer Fw (20 pmol/µL)	2 µL
Primer Rv (20 pmol/µL)	2 µL
Water PCR	9.5 µL
dNTPs (10 nM)	2.5 µL
MgCl_2_	3 µL

**Table 4:** Thermocycler program for cyt *b* gene of skunks.

Thermocycler	Program
Step	
1 cycle	3 min 94 °C
39 cycles	30 seg 94 °C
39 cycles	15 seg 60 °C
39 cycles	30 seg 72 °C
1 cycle	7 min 72 °C
∞	4 °C

## Data Availability

GenBank access numbers for all the sequences generated in this study have been shown in Table 1: PQ963930, PQ963931, PQ963932, PV012461, PV012462, PV012463, PV012464, PV012465, PV012466, PV012467, PV012468.

## Supplementary Materials

The following supporting information can be downloaded at: https://www.mdpi.com/article/10.3390/microorganisms13020262/s1. Table S1: GenBank accession numbers of sequences used in the phylogenetic analyses of rabies virus from the Americas.
