# Supplementary material for: The Role of Skunks in the Epidemiology of Rabies in the State of Yucatan from 2000 to 2022: Current Perspectives and Future Research Directions
Source: Microorganisms. 2025 Jan 25;13(2):262. doi: 10.3390/microorganisms13020262 (PMC11857469; doi:10.3390/microorganisms13020262)
Supplement: Supplementary file 1 [file microorganisms-13-00262-s001.zip › microorganisms-3333287-supplementary material-update.pdf]

Supplementary Table S1. GenBank accession numbers of sequences used in the phylogenetic analyses of rabies virus from the Americas.

#	GenBank Accesion No.	Location	#	GenBank Accesion No.	Location	#	GenBank Accesion No.	Location
1	AY561805	Mexico	31	KU550101	Costa Rica	61	FJ228485	Mexico
2	AY561806	Mexico	32	AY877435	Mexico	62	FJ228489	Mexico
3	AY561804	Mexico	33	OM971003	Mexico	63	FJ228492	El Salvador
4	OM203040	USA	34	OM971004	Mexico	64	FJ228494	Mexico
5	GU991834	Mexico	35	OM971005	Mexico	65	FJ228496	Puerto Rico
6	GU991823	Mexico	36	KX708499	Mexico	66	FJ228497	Puerto Rico
7	GU991824	Mexico	37	KX708500	Mexico	67	FJ228498	Peru
8	GU991825	Mexico	38	KX708501	Mexico	68	FJ228499	Peru
9	GU991826	Mexico	39	GU644773	USA	69	FJ228500	Peru
10	GU991827	Mexico	40	AF394875	USA	70	FJ228501	Peru
11	GU991828	Mexico	41	GU644776	USA	71	FJ228503	Mexico
12	GU991829	USA	42	AY854594	Mexico	72	FJ228507	Mexico
13	GU991830	Mexico	43	AY854592	Mexico	73	FJ228508	Mexico
14	GU991831	USA	44	KF656696	Guatemala	74	FJ228510	Mexico
15	GU991833	Mexico	45	JQ685936	Mexico	75	FJ228511	Mexico
16	GU991835	USA	46	AY561810	Mexico	76	FJ228512	Mexico
17	GU991836	USA	47	JX856024	USA	77	FJ228517	Honduras
18	GU991837	USA	48	JX856026	USA	78	FJ228523	Mexico
19	GU991838	USA	49	JX856016	USA	79	FJ228524	Mexico
20	GU991843	USA	50	JX855980	USA	80	FJ228525	Mexico
21	GU991844	Mexico	51	MW055087	USA	81	FJ228527	Mexico
22	GU991845	Mexico	52	HM113792	USA	82	FJ228544	Mexico
23	GU991846	Mexico	53	HM113762	USA	83	FJ228545	Mexico
24	GU991847	Mexico	54	HM113751	USA	84	GU991839	USA
25	JQ685963	Mexico	55	HM113707	USA	85	GU991840	USA
26	KJ001528	Mexico	56	HM113690	USA	86	GU991842	USA
27	OM971002	Mexico	57	HM113688	USA	87	KY688151	Finland
28	KJ001526	Mexico	58	FJ228533	Mexico	88	**203MxYucSkunk2020**	**Mexico**
29	KJ001527	Mexico	59	FJ228483	Mexico	89	**204MxYucSkunk2020**	**Mexico**
30	OM971001	Mexico	60	FJ228484	Mexico	90	**482MxYucSkunk2019**	**Mexico**
